# Prediction Model for Postoperative Quality of Life Among Breast Cancer Survivors Along the Survivorship Trajectory From Pretreatment to 5 Years: Machine Learning–Based Analysis

**DOI:** 10.2196/45212

**Published:** 2023-08-24

**Authors:** Danbee Kang, Hyunsoo Kim, Juhee Cho, Zero Kim, Myungjin Chung, Jeong Eon Lee, Seok Jin Nam, Seok Won Kim, Jonghan Yu, Byung Joo Chae, Jai Min Ryu, Se Kyung Lee

**Affiliations:** 1 Department of Clinical Research Design and Evaluation SAIHST Sungkyunkwan University Seoul Republic of Korea; 2 Center for Clinical Epidemiology Samsung Medical Center Seoul Republic of Korea; 3 Medical AI Research Center Samsung Medical Center Seoul Republic of Korea; 4 Department of Surgery Samsung Medical Center Sungkyunkwan University School of Medicine Seoul Republic of Korea

**Keywords:** breast cancer survivor, quality of life, machine learning, trajectory, predict, develop, breast cancer, survivor, cancer, oncology, algorithm, model, QoL

## Abstract

**Background:**

Breast cancer is the most common cancer and the most common cause of cancer death in women. Although survival rates have improved, unmet psychosocial needs remain challenging because the quality of life (QoL) and QoL-related factors change over time. In addition, traditional statistical models have limitations in identifying factors associated with QoL over time, particularly concerning the physical, psychological, economic, spiritual, and social dimensions.

**Objective:**

This study aimed to identify patient-centered factors associated with QoL among patients with breast cancer using a machine learning (ML) algorithm to analyze data collected along different survivorship trajectories.

**Methods:**

The study used 2 data sets. The first data set was the cross-sectional survey data from the Breast Cancer Information Grand Round for Survivorship (BIG-S) study, which recruited consecutive breast cancer survivors who visited the outpatient breast cancer clinic at the Samsung Medical Center in Seoul, Korea, between 2018 and 2019. The second data set was the longitudinal cohort data from the Beauty Education for Distressed Breast Cancer (BEST) cohort study, which was conducted at 2 university-based cancer hospitals in Seoul, Korea, between 2011 and 2016. QoL was measured using European Organization for Research and Treatment of Cancer QoL Questionnaire Core 30 questionnaire. Feature importance was interpreted using Shapley Additive Explanations (SHAP). The final model was selected based on the highest mean area under the receiver operating characteristic curve (AUC). The analyses were performed using the Python 3.7 programming environment (Python Software Foundation).

**Results:**

The study included 6265 breast cancer survivors in the training data set and 432 patients in the validation set. The mean age was 50.6 (SD 8.66) years and 46.8% (n=2004) had stage 1 cancer. In the training data set, 48.3% (n=3026) of survivors had poor QoL. The study developed ML models for QoL prediction based on 6 algorithms. Performance was good for all survival trajectories: overall (AUC 0.823), baseline (AUC 0.835), within 1 year (AUC 0.860), between 2 and 3 years (AUC 0.808), between 3 and 4 years (AUC 0.820), and between 4 and 5 years (AUC 0.826). Emotional and physical functions were the most important features before surgery and within 1 year after surgery, respectively. Fatigue was the most important feature between 1 and 4 years. Despite the survival period, hopefulness was the most influential feature on QoL. External validation of the models showed good performance with AUCs between 0.770 and 0.862.

**Conclusions:**

The study identified important factors associated with QoL among breast cancer survivors across different survival trajectories. Understanding the changing trends of these factors could help to intervene more precisely and timely, and potentially prevent or alleviate QoL-related issues for patients. The good performance of our ML models in both training and external validation sets suggests the potential use of this approach in identifying patient-centered factors and improving survivorship care.

## Introduction

Breast cancer is the most common cancer and the most common cause of cancer death in women worldwide [1]. In the past years, breast cancer prognosis has significantly improved over time. Currently, the 5-year survival rates are in the range of 90%, and 10-year survival is about 80%. Given the increase in survival, a survivorship care plan is necessary over time, with particular attention to the quality of life (QoL) [2]. However, for many survivors, cancer survivorship is characterized by uncertainty regarding follow-up care and unmet psychosocial needs [3].

To develop tailored interventions and to provide appropriate survivorship care, it is necessary to find predictors for QoL during different phases of survivorship [4,5]. Although some predictors for QoL have been identified in several studies [6], almost all focused on 1 specific predictor. Fewer models have made individual predictions on QoL due to the complexity of clinical profiles and the inability to consider relevant interactions a priori. In addition, according to a recent cohort study, the QoL and the QoL-related factors change over time [7]. However, it is difficult to generate those models using traditional statistical methods.

To overcome the limitation of traditional models, a few machine learning (ML) models have been proposed in the literature that predict the QoL of breast cancer survivors. However, there were only a few ML models for QoL prediction with limitations [6,8-12]. First, most models did not fully include multidimensional factors. Although some studies included patient-centered factors such as functional impairment and psychological symptoms, they still missed key variables for the QoL of long-term survivors, such as spiritual well-being [13-16]. Second, only a few studies examined the predictors of QoL for long-term survivors. Third, QoL-related factors are known to change over time due to their multilayer and multidimensional characteristics [2], but previous models did not identify predictors as time varying. Fourth, the previous prediction models for QoL were difficult to interpret, and their overall prediction values were limited. Recently, it is possible to develop an ML algorithm that allows for interpretation [17]. Thus, this study aimed to identify patient-centered factors associated with QoL using an ML algorithm to analyze data from a cohort of Korean patients with breast cancer along different survivorship trajectories.

## Methods

### Study Population and Design

To produce a robust tool to identify factors associated with QoL during different survival phases, 2 different data sets were used. These included (1) the cross-sectional survey data from the Breast Cancer Information Grand Round for Survivorship (BIG-S) study to develop a model and (2) the longitudinal cohort data from the Beauty Education for Distressed Breast Cancer (BEST) cohort study to validate the model.

### Development Set

The BIG-S study recruited consecutive breast cancer survivors (BCS) who visited the outpatient breast cancer clinic at the Samsung Medical Center in Seoul, Republic of Korea, between November 2018 and April 2019. The BIG-S study included survivors aged over 20 years and who did not have secondary cancer, metastasis, or recurrence. A total of 6265 survivors agreed to participate in the BIG-S study: before surgery (n=1980) and 1 year (n=653), 2 years (n=1265), 3 years (n=921), 4 years (n=682), and 5 years (n=764) after surgery.

### External Validation Set

The BEST study (n=432) was conducted at 2 university-based cancer hospitals in Seoul, Republic of Korea, to evaluate the effect of cancer treatment-induced altered body image and QoL. Subjects were eligible to participate if they were between 18 and 65 years of age, had a diagnosis of breast cancer (ductal carcinoma in situ, stages I-III), had no sign of metastasis, were expected to have breast cancer surgery, and did not receive preoperative chemotherapy or radiation therapy [15]. There were 323 patients before surgery, and 297, 215, 214, and 232 patients who were followed prior to surgery and at 1, 2, 3, and 5 years following surgery, respectively.

### Measures

In this study, the target variable was poor QoL, which was measured using a 7-point Likert scale with the European Organization for Research and Treatment of Cancer (EORTC) QoL Questionnaire Core 30 questionnaire. The single item has been validated to measure overall QoL [18].

To determine the factors associated with QoL, information about sociodemographics; diagnosis and treatment; and physical, psychological, social, and spiritual well-being was included based on a literature review (Table S1 in Multimedia Appendix 1) [5,15]. Sociodemographic factor data, including education level, marital status, monthly house income, working status during the survey, drinking status, and smoking status, were obtained using a standard questionnaire. Diagnosis and treatment data were obtained from electronic medical records. These data included types of operations, locations of tumors, comorbidities, laboratory test results, pathology stage, and type of treatment (chemotherapy, hormone therapy, target therapy, and radiotherapy).

In patient-reported outcomes, we followed the recommendation from International Consortium for Health Outcomes Measurement. To measure physical, psychological, and social well-being, the EORTC QoL Questionnaire Core 30 and Breast Cancer-Specific Module were used, and related symptoms and functions were evaluated. These included fatigue, pain, nausea and vomiting, emotional function, body image, social function, and role functioning. Spiritual well-being was evaluated using 3 questions from the Spiritual Well-being Domain of the Korean version of QoL of Cancer Survivors questionnaire [19]. In order to measure menopause symptoms, the Menopause Rating Scale (MRS) was used. The MRS included 11 items in 3 dimensions, including somatic-vegetative, psychological, and urogenital. The composite scores (score range 0-44) were based on adding the scores of the items from the respective dimensions.

### Statistical Analysis

**Figure 1:**
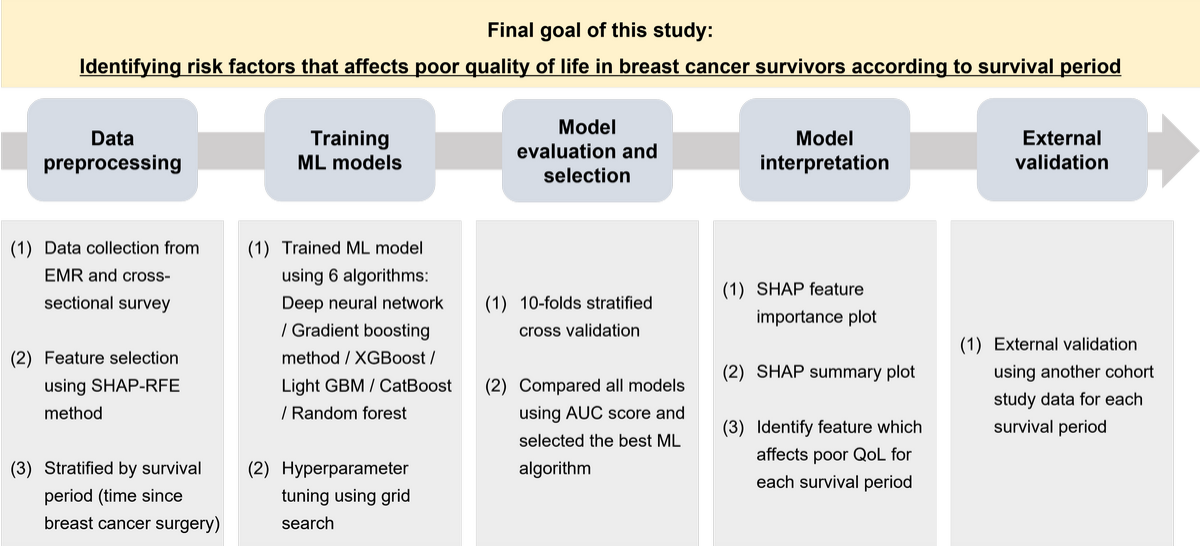
Workflow of machine learning. AUC: area under the curve; EMR: electronic media record; ML: machine learning; QoL: quality of life; SHAP: Shapley Additive Explanations; SHAP-RFE: shapley additive explanations-recursive feature elimination.

This study was conducted in five steps: (1) data preprocessing, (2) training ML models, (3) model evaluation and selection, (4) model interpretation, and (5) external validation (Figure 1). The target variable of “poor QoL” was defined as a score lower than 66 on the global health status scale (range 0-100). Factors associated with QoL were selected from the BIG-S data set. Since some of the treatment-related variables were not in the data collected prior to surgery, 37 and 45 features were selected for data preprocessing from variables collected before surgery and after surgery, respectively. In all algorithms, missing values were forward filled with the closest observation. If no past value was present, the training set mean was imputed by matching the participants’ ages and pathology stages.

To train the ML models, the feature selection method and the recursive feature elimination method based on the Shapley Additive Explanation (SHAP) method were used to reduce the model complexity and to remove unnecessary features that generate noise in the prediction model. The SHAP method is one of the explainable artificial intelligence methods [20]. Through the Shapley values obtained using the SHAP method, how much a variable affects the outcome prediction and how the variable affects the outcome in each instance can be observed. For model evaluation and selection, we compared the performance of 6 different algorithms, including the deep neural network, gradient boosting machine, XGBoost, light gradient boosting machine, CatBoost, and random forest. For training the models, the grid search method was used for hyperparameter tuning. Hyperparameters are parameters that directly affect the learning process of the model and are determined by the user to improve model performance and avoid overfitting. After specifying the possible value range of hyperparameters for each model (Table S2 in Multimedia Appendix 1), models were trained using all possible combinations of hyperparameters, and then the optimal combinations were selected.

To validate and evaluate the model, 10-fold stratified cross-validation was used. The entire training data set was divided into 10-fold equal size subsamples by stratifying for the outcome variables. A single subsample was retained as the validation data for testing the model, and the remaining 9 subsamples were used as training data and the process was repeated 10 times. Using the 10 cross-validation results, the area under the receiver operating characteristic curve (AUC) scores were averaged for each model, and the final model with the highest mean AUC was selected. In this study, we also used SHAP values to interpret feature contributions and assess the clinical significance of predictive models. According to a previous study, the SHAP value is the measurement of the marginal contribution of each feature in different combinations. The SHAP value of a feature can be interpreted as the difference between the model’s predicted value when that feature is included versus when it is excluded, taking into account all possible combinations of other features. The base value, on the other hand, is the average predicted value of the model for all samples. When the SHAP value of a feature is positive, it means that including that feature has a positive effect on the predicted value, while a negative SHAP value indicates a negative effect. Overall, SHAP values help to explain how each feature contributes to the model’s predictions, providing insight into the model’s decision-making process [21,22].

Finally, external validation was confirmed using the BEST cohort data set, which was a completely different data set from that used for model training. The poor QoL group was predicted by inputting the external validation data set into the final model that trained the entire training data set using the ML algorithm selected by the survival period. It is notable that between the 3- and 4-year models, there was no validation data set because there was no participant follow-up within the BEST cohort for these time periods. The validation performances were also evaluated using AUC, accuracy, F1 score, sensitivity, and specificity, and were also compared with training performance.

All analyses were performed in the Python 3.7 programming environment (Python Software Foundation) and used the scikit-learn package and TensorFlow Keras framework.

### Ethics Approval

The study was approved by the institutional review board of the Samsung Medical Center, Seoul, Republic of Korea, in the development set (SMC-2018-08-070) and external validation set (SMC-2011-07-019). Informed consent was obtained from all study participants.

## Results

### Characteristics of Participants

All 6265 participants were included in the full analysis data set. The mean age of the study participants was 50.6 (SD 8.66) years and 46.8% (n=2004) of participants were stage 1. In the training data set, 48.3% (n=3026) of the participants were classified into the poor QoL group (Table 1). The proportion of patients with breast cancer with poor QoL was 67.4% (n=1335) at diagnosis, and 41.8% (n=273), 39.3% (n=497), 40.1% (n=369), 36.1% (n=246), and 40.1% (n=306) patients had poor QoL at 1, 2, 3, 4, and 5 years after surgery, respectively (Table 1).

**Table 1:** Characteristics of participants.

Characteristics		Preoperation (n=1980)	Within 1 year (n=653)	Between 1 and 2 years	Between 2 and 3 years (n=921)	Between 3 and 4 years (n=682)	Between 4 and 5 years (n=764)	*P* value	
Age (years), mean (SD)		50.54 (8.74)	49.12 (9.10)	49.78 (8.62)	50.96 (8.68)	50.29 (8.39)	52.37 (8)	<.001	
**BMI (kg/m^2^), n (%)**									.001
	Underweight (<18.5)	110 (5.6)	34 (5)	56 (4)	45 (5)	25 (4)	36 (5)		
	Normal (18.5-23)	985 (49.7)	352 (53.9)	675 (53.4)	506 (54.9)	392 (57.5)	416 (54.5)		
	Overweight (23-25)	385 (19.4)	137 (21)	281 (22.2)	202 (21.9)	134 (19.6)	156 (20.4)		
	Obese (≥25)	500 (25.3)	130 (19.9)	253 (20)	168 (18.2)	131 (19.2)	156 (20.4)		
**Education, n (%)**									<.001
	Middle school or lower	103 (5.2)	58 (8.9)	88 (7)	68 (7.4)	65 (9.5)	82 (10.7)		
	High school	324 (16.4)	217 (33.2)	445 (35.2)	315 (34.2)	223 (32.7)	271 (35.5)		
	University graduates or higher	1553 (78.4)	378 (57.9)	732 (57.9)	538 (58.4)	394 (57.8)	411 (53.8)		
**Working status at survey, n (%)**									<.001
	Working	868 (43.8)	371 (56.8)	713 (56.4)	524 (56.9)	366 (53.7)	417 (54.6)		
	Not working	1112 (56.2)	282 (43.2)	552 (43.6)	397 (43.1)	316 (46.3)	347 (45.4)		
**Marital status at survey, n (%)**									.70
	Single	185 (9.3)	61 (9.3)	101 (8)	68 (7.4)	67 (9.8)	55 (7.2)		
	Married	1617 (81.7)	541 (82.8)	1043 (82.5)	764 (83)	554 (81.2)	636 (83.2)		
	Divorced	118 (6)	31 (4.7)	75 (5.9)	52 (5.6)	37 (5.4)	42 (5.5)		
	Bereavement	60 (3)	20 (3.1)	46 (3.6)	37 (4)	24 (3.5)	31 (4.1)		
**Monthly family income (US $), n (%)**									.001
	≤$2000	479 (24.2)	142 (21.7)	284 (22.5)	219 (23.8)	154 (22.6)	187 (24.5)		
	$2000-$4000	636 (32.1)	205 (31.4)	364 (28.8)	246 (26.7)	163 (23.9)	218 (28.5)		
	>$4000	865 (43.7)	306 (46.9)	617 (48.8)	456 (49.5)	365 (53.5)	359 (47)		
**Drinking status, n (%)**									
	Never	977 (49.3)	314 (48.1)	598 (47.3)	433 (47)	307 (45)	369 (48.3)		
	Past	689 (34.8)	287 (44)	500 (39.5)	324 (35.2)	225 (33)	195 (25.5)		
	Current	314 (15.9)	52 (8)	167 (13.2)	164 (17.8)	150 (22)	200 (26.2)		
**Smoking status, n (%)**									<.001
	Never smoker	1789 (90.4)	602 (92.2)	1137 (89.9)	850 (92.3)	633 (92.8)	710 (92.9)		
	Ever smoker	191 (9.6)	51 (8)	128 (10.1)	71 (7.7)	49 (7)	54 (7)		
Comorbidity (yes), n (%)		672 (33.9)	235 (36)	485 (38.3)	373 (40.5)	285 (41.8)	350 (45.8)	<.001	
Physical activity (yes), n (%)		688 (34.7)	579 (88.7)	1138 (90)	802 (87.1)	601 (88.1)	665 (87)	<.001	
**Pathology stage, n (%)**									<.001
	0 or CR (NRT)	—^a^	119 (18.2)	212 (16.8)	141 (15.3)	78 (11)	68 (9)		
	I	—	321 (49.2)	573 (45.3)	419 (45.5)	309 (45.3)	382 (50)		
	II	—	181 (27.7)	391 (30.9)	292 (31.7)	223 (32.7)	248 (32.5)		
	III or IV	—	32 (5)	89 (7)	69 (7.5)	72 (10.6)	66 (8.6)		
**Type of surgery, n (%)**									<.001
	Mastectomy with reconstruction	—	112 (17.2)	213 (16.8)	172 (18.7)	119 (17.4)	95 (12.4)		
	Mastectomy without reconstruction	—	112 (17.2)	198 (15.7)	136 (14.8)	150 (22)	165 (21.6)		
	Breast conservation surgery	—	429 (65.7)	854 (67.5)	613 (66.6)	413 (60.6)	504 (66)		
Chemotherapy (yes), n (%)		—	160 (24.5)	419 (33.1)	365 (39.6)	305 (44.7)	389 (50.9)	<.001	
Radiation therapy (yes), n (%)		—	482 (73.8)	968 (76.5)	675 (73.3)	496 (72.7)	580 (75.9)	.24	
Hormone therapy (yes), n (%)		—	505 (77.3)	981 (77.5)	721 (78.3)	534 (78.3)	618 (80.9)	.44	
Target therapy (yes), n (%)		—	84 (13)	186 (14.7)	140 (15.2)	98 (14.4)	116 (15.2)	.72	

^a^Not available.

In the validation set, the mean age was 46.5 (SD 7.87) years, and 47.1% (n=428) of the participants were stage 1 (Table S3 in Multimedia Appendix 1). Compared to the training set, patients in the external validation set were relatively younger. Among these participants, 48.6% (n=573) were classified as having poor QoL. Patients with poor QoL prior to surgery and 1, 2, 3, and 5 years after surgery made up 70.4% (n=100), 53.2% (n=255), 49.4% (n=79), 48.5% (n=95), and 33.8% (n=72) of the groups, respectively (Table S4 in Multimedia Appendix 1).

### Performances of Machine Learning Models for Each Survival Period

The available features in the training data set were used to build QoL prediction models based on 6 ML algorithms (Table S4 in Multimedia Appendix 1). From the whole data set, between 9 and 16 features were selected using the SHAP-RFE method. The AUC values of 6 ML algorithms were all over 0.75. Among 6 ML algorithms associated with the survival periods, all the final models were over 0.8 (Table S5 in Multimedia Appendix 1). The best predictive performances were observed using the CatBoost algorithm for all survival periods: overall (AUC 0.823), baseline (AUC 0.835), within 1 year (AUC 0.860), between 2 and 3 years (AUC 0.808), between 3 and 4 years (AUC 0.820), and between 4 and 5 years (AUC 0.826) (Table 2). All 5 model evaluation metric averages calculated through 10-fold stratified cross-validation for each survival period were higher than 0.7 and the AUC exceeded 0.8 (0.804-0.860), showing that the ML models performed well.

**Table 2:** Performance metrics by survival period.

Survival period	Overall^a^	Baseline^a^	Within 1 years^a^	Between 1 and 2 years^b^	Between 2 and 3 years^a^	Between 3 and 4 years^a^	Between 4 and 5 years^a^
AUC^c^	0.823	0.835	0.860	0.804	0.808	0.820	0.826
Accuracy	0.756	0.774	0.818	0.765	0.767	0.783	0.793
F1 score	0.707	0.817	0.782	0.705	0.709	0.723	0.752
Sensitivity	0.749	0.753	0.787	0.722	0.721	0.792	0.782
Specificity	0.761	0.815	0.839	0.793	0.797	0.777	0.801

^a^Observed using CatBoost algorithm.

^b^Observed using a gradient boosting algorithm.

^c^AUC: area under the curve.

### Important Features for Each Survival Period

**Figure 2:**
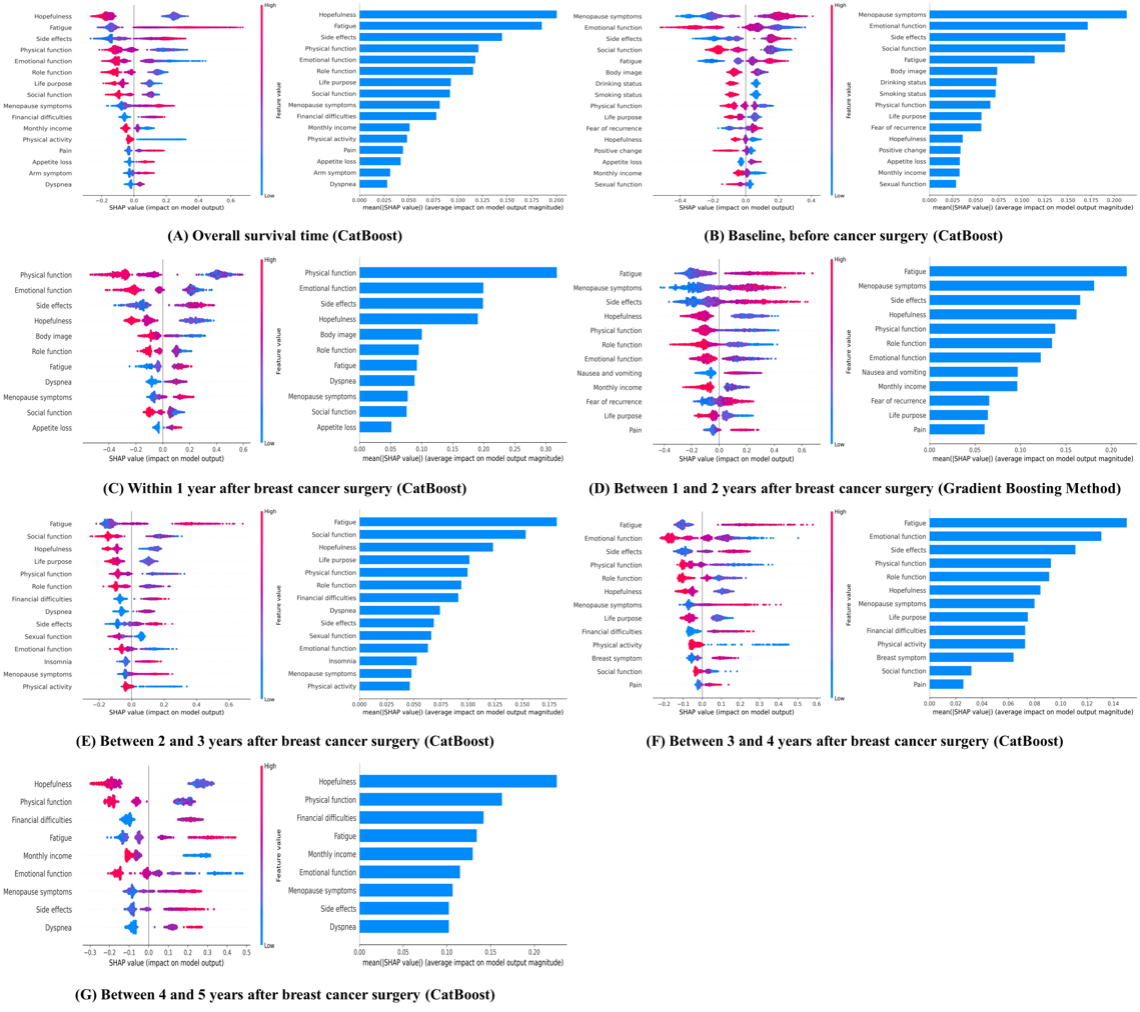
Summary plot of Shapley Additive Explanation.

The most important prognostic features for each survival period were identified using the feature importance from the SHAP method (Figure 2).

**Figure 3:**
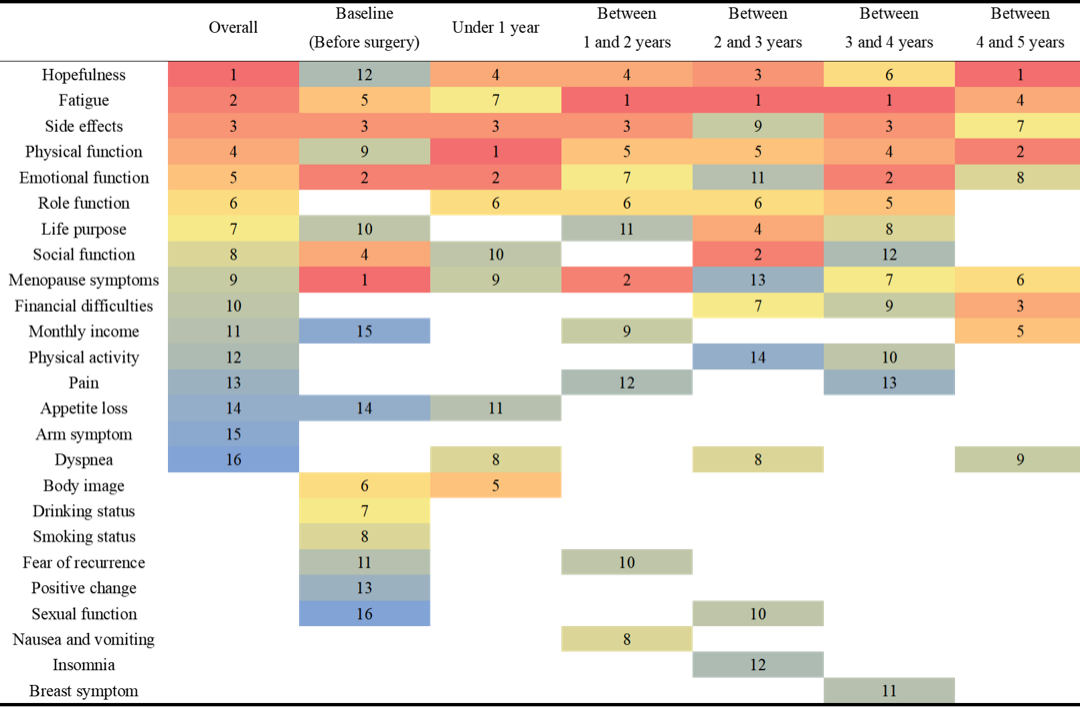
Rank of feature obtained by Shapley Additive Explanation value.

Regardless of survival period, hopefulness (SHAP value 0.2005) was the most important feature, and fatigue, side effects, physical function, emotional function, and role function were also important features. By the survival period, menopause symptoms (SHAP value 0.2137) and emotional function (SHAP value 0.1715) were the most important features prior to breast cancer surgery (Table S6 in Multimedia Appendix 1). For the within 1-year period, physical function (SHAP value 0.3177) was the most important feature, followed by emotional function, side effects, hopefulness, and body image. For the periods between 1-2, 2-3, and 3-4 years, fatigue (SHAP values 0.2172, 0.1819, and 0.1503, respectively) was the most important feature, followed by menopause symptoms, social function, and emotional function. For the period between 4 and 5 years, hopefulness (SHAP value 0.2370) was the most important feature, followed by physical function, dyspnea, financial difficulties, monthly income, menopause symptoms, side effects, and emotional function (Figure 3).

### External Validation

All 5 model evaluation metric averages calculated in the external validation set were higher than 0.7 (Table 3).

**Figure 4:**
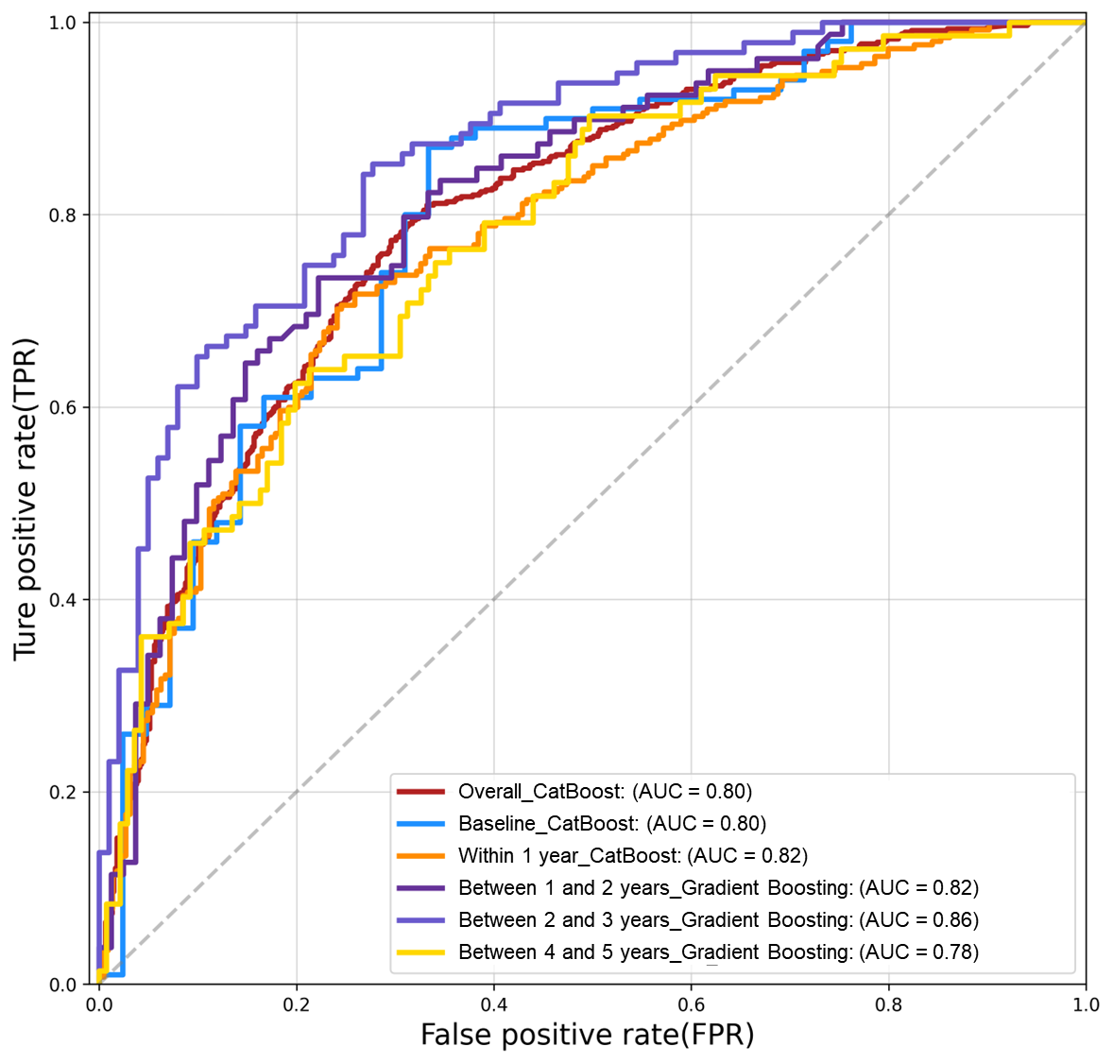
Receiver operating characteristics curve. AUC: area under the curve.

For external validation, the receiver operating characteristic curves for each survival period were used to calculate the AUC. When the final trained models for each survival period were externally validated using the BEST data set, the validation AUC was between 0.770 and 0.862, and the differences from the results of the 10-fold stratified cross-validation for training were from 0.009 to 0.056 (Figure 4).

**Table 3:** Performance metrics for external validation for survival period.

Survival period	Overall^a^	Baseline^a^	Within 1 years^a^	Between 1 and 2 years^b^	Between 2 and 3 years^a^	Between 4 and 5 years^a^
AUC^c^	0.800	0.799	0.778	0.816	0.863	0.779
Accuracy	0.810	0.737	0.729	0.756	0.786	0.742
F1 score	0.866	0.750	0.734	0.748	0.794	0.621
Sensitivity	0.870	0.810	0.702	0.734	0.853	0.625
Specificity	0.667	0.668	0.759	0.778	0.723	0.801

^a^Observed using CatBoost algorithm.

^b^Observed using a gradient boosting algorithm.

^c^AUC: area under the curve.

## Discussion

### Principal Findings

In this study, we developed and validated factors associated with QoL including physical, psychological, economic, spiritual, and social dimensions by survivorship trajectory using an ML algorithm. The developed model and external validation model performance were good for all survival trajectories. Before surgery, menopause symptoms and emotional function were important features. Within 1 year after the surgery period, physical function was the most important feature. Between 1 and 4 years, fatigue was the most important feature. Regardless of the survival period, hopefulness was the most influential feature of spiritual well-being.

In this study, the AUC for evaluating model performances surpassed 0.8 for all survival periods, and the results of external validation using data collected in other studies were also greater than 0.77. This performance is much better than that of previous studies that predicted QoL using ML modeling, which reported values ranging from 0.476 to 0.793 [6,9-12]. These ML-based breast cancer QoL prediction models were developed with not only clinical and sociodemographic factors but also with the integration of information from multiple factors, thus ensuring better model performance. Furthermore, this study stratified the model by time periods following surgery and found that there were different factors associated with QoL during each time period.

Prior to breast cancer surgery, menopause symptoms and emotional function were selected as important features that affect QoL in BCS. Among menopause symptoms, the most important factor was in the psychological domain, which included depressive mood, irritability, and anxiety. According to a previous study, depression and anxiety are the 2 most common psychiatric comorbidities encountered in patients with breast cancer [6,7]. Patients with breast cancer may experience depression or anxiety at any stage of their illness, from prediagnosis to the terminal phase of the illness. Studies in Western countries have shown that the prevalence of depression ranges from 1% to 56%, whereas the prevalence of depression found in Asian studies was between 12.5% and 31% [23]. Thus, timely psychosocial care should be needed for newly diagnosed distressed patients with cancer.

In this study, physical function was the most important feature that affected QoL in the group that was within 1 year after breast cancer surgery. This result was consistent with previous reports that BCS are susceptible to physical functioning–related problems and often experience treatment-related declines in their physical functioning capabilities within the 1-year period following their cancer diagnosis [24]. Treatment-related systemic side effects that occur after completion of treatment affect physical function, and poor physical function negatively affects the QoL of BCS [25]. Furthermore, physical functioning–related problems may persist even after treatment has been completed [26]. Persistent physical symptoms can increase fatigue and hinder patients’ return to normal life, thereby reducing their QoL. Future research should focus on the development and testing of interventions for managing physical function in order to improve the QoL of patients with breast cancer.

Between 1 and 4 years after breast cancer surgery, fatigue is the most important feature that affects QoL in BCS. Cancer-related fatigue is one of the most distressing and common posttreatment sequelae among survivors of early-stage breast cancer [27]. More than 30% of patients with breast cancer experience persistent fatigue symptomatology up to 10 years after completion of treatment [28]. Cancer-related fatigue can result in substantial adverse physical, psychosocial, and socioeconomic consequences and has a negative impact on overall QoL. For BCS 1 year after diagnosis, reducing the burden of fatigue might be a preferable approach to improve their QoL and focusing on fatigue symptoms can help to enhance the long-term survivors’ QoL [29]. Cancer-related fatigue is considered a complex symptom, with multidimensional and intricate aspects. The existence of physical, psychological, and emotional disturbance has been proven [30], and numerous evidence-based interventions for the management of fatigue have been recommended [31], most of them being complex nonpharmacological interventions. In order to address all dimensions of fatigue, nonpharmacological interventions should be tested and assessed.

Hopefulness was the most important feature in all survival period models, especially, between 4 and 5 years. Spiritual well-being was a predictor of improved QoL and is one of the important outcomes to measure in BCS [32]. Previous studies have indicated that survivors who had more hope in their lives were more likely to have better QoL [33]. Hope could help patients find a sense of health in the midst of disease to cope with various cancer symptoms and fear of recurrence and to find meaning and peace of mind [34]. These positive effects of hope might also improve QoL in BCS. Therefore, patient-centered interventions that help survivors find purpose in life by focusing on themes such as planning for life after cancer and value-based sources of meaning to have hope should be provided.

In this study, we performed an external validation to test the generalizability of our models, which is a strength compared to the previous study that did not perform external validation [6,9-12]. This aspect is important as it demonstrates the effectiveness of our models and their potential to be applied to other settings. Through external validation, we could assess our models’ robustness and confirm their ability to provide accurate predictions in new and independent data sets. This enhances the reliability and use of our models, and highlights the potential of ML approaches in improving survivorship care for patients with breast cancer.

This study has several limitations. First, it is a cross-sectional study, and the directions of the associations between QoL; symptoms; and physical, psychosocial, and spiritual functions could be interchangeable. In fact, patients who had a poor QoL might report poorer function status. Second, QoL was measured using a single item from the EORTC-C30, and this might not be a reliable method to measure an individual’s QoL. However, this single question has been validated to measure a person’s overall QoL, and it has been widely used in different cultures and countries, including Korea. Lastly, the results of our study might not be generalizable to other cancer survivors in other settings. Further studies with various types of cancer survivors are necessary to confirm the study findings and its generalizability.

Despite these limitations, this study had several strengths. First, we included physical, psychological, economic, spiritual, and social dimensions and clinical factors. Second, we developed a prediction model to predict QoL from pretreatment to 5 years after surgery. Third, we developed different ML-based QoL surveillance models across survivorship. Fourth, we used SHAP methods, which allow for the interpretation of the model by the reader. Fifth, we performed external validation and the models showed good performance.

### Conclusions

The results of this study may provide valuable information on developing a patient-centered survival care plan. Understanding the changing trends of influencing factors associated with QoL during different survival trajectories could help health care professionals intervene timely and appropriately in order to prevent or alleviate factors more precisely.

## Appendix

Multimedia Appendix 1 Supplementary tables.

## Abbreviations

AUC: area under the curve
BCS: breast cancer survivor
BEST: Beauty Education for Distressed Breast Cancer
BIG-S: Breast Cancer Information Grand Round for Survivorship
EORTC: European Organization for Research and Treatment of Cancer
ML: machine learning
MRS: Menopause Rating Scale
QoL: quality of life
SHAP: Shapley Additive Explanation

## Data Availability

The data supporting this study’s findings are available on request from the corresponding author (SKL). The data are not publicly available because they contain information that could compromise research participant consent.
