# Supplementary material for: Prediction Model for Postoperative Quality of Life Among Breast Cancer Survivors Along the Survivorship Trajectory From Pretreatment to 5 Years: Machine Learning–Based Analysis
Source: JMIR Public Health Surveill. 2023 Aug 24;9:e45212. doi: 10.2196/45212 (PMC10485708; doi:10.2196/45212)
Supplement: Multimedia Appendix 1 [file publichealth_v9i1e45212_app1.docx]

| **Table S1**. Features used for model training. | |
| --- | --- |
| Type of variables | Contents |
| Clinical data | Comorbidity  Location of breast cancer  Pathology stage  Type of breast cancer surgery  Chemotherapy  Radiation therapy  Hormone therapy  Target therapy |
| Sociodemographic data | Age  Times since breast cancer surgery  Body Mass Index (BMI)  Education levels  Working status at survey  Marital status at survey  Monthly family income  Drinking status  Smoking status  Physical activity |
| Laboratory test data | WBC  RBC  Hemoglobin  Platelet  Eosinophil  Basophil  Lymphocyte  Albumin  fasting glucose  cholesterol  ALT  Creatinine  CEA NR1102  CA 15.3 NR1105 |
| Patient reported outcome data | Physical (physical function, fatigue, pain nausea and vomiting diarrhea, constipation, appetite loss, sleep disturbance, dyspnea, arm symptoms, systemic therapy side effects, hair loss,  menopause symptoms) |
|  | Psychological (emotional function and fear of recurrence) |
|  | social (role function, social function, financial difficulties) |
|  | Spiritual (uncertainty, positive change, life purpose, hopefulness) |

| **Table S2.** Hyperparameters. | | |
| --- | --- | --- |
| **Survival period** | **Algorithm** | **Hyper parameters** |
| Overall | CatBoost | - Bagging temperature: 0.6  - Depth: 7  - Iteration: 50  - l2 leaf reg: 1  - Learning rate: 0.03 |
| Baseline (before surgery) | CatBoost | - Bagging temperature: 0.6  - Depth: 7  - Iteration: 50  - l2 leaf reg: 1  - Learning rate: 0.03 |
| Under 1 year | CatBoost | - Bagging temperature: 0.6  - Depth: 6  - Iteration: 50  - l2 leaf reg: 1  - Learning rate: 0.03 |
| Between 1 and 2 years | Gradient Boosting Machines | - Learning rate: 0.03  - Max depth: 5  - Min impurity decrease: 0  - Min sample leaf: 50  - Min samples split: 20  - N estimators: 50  - Subsample = 1.0 |
| Between 2 and 3 years | CatBoost | - Bagging temperature: 0.6  - Depth: 7  - Iteration: 50  - l2 leaf reg: 2  - learning rate: 0.03 |
| Between 3 and 4 years | CatBoost | - Bagging temperature: 0.6  - Depth: 7  - Iteration: 50  - l2 leaf reg: 3  - Learning rate: 0.03 |
| Between 4 and 5 years | CatBoost | - Bagging temperature: 0.6  - Depth: 7  - Iteration: 50  - l2 leaf reg: 2  - Learning rate: 0.03 |

| **Table S3.** Characteristic of validation set. | | | | | | | |
| --- | --- | --- | --- | --- | --- | --- | --- |
| **Characteristic** | **Overall survival** | **Baseline** | **1 year** | **2 years** | **3 years** | **5 years** | ***P* value** |
|  | *n* = 1,178 | *n* = 142 | *n* = 479 | *n* = 160 | *n* = 196 | *n* = 213 |  |
| **Age** | 46.40 (7.75) | 45.80 (7.70) | 46.50 (7.89) | 45.89 (7.62) | 46.82 (7.62) | 46.00 (7.41) | 0.749 |
| **BMI** |  |  |  |  |  |  | 0.998 |
| Underweight (<18.5 kg/m^2^) | 35 (3.0) | 6 (4.2) | 15 (3.1) | 6 (3.8) | 6 (3.1) | 6 (2.8) |  |
| Normal (18.5–23.0 kg/m^2^) | 615 (52.2) | 77 (54.2) | 247 (51.6) | 87 (54.4) | 101 (51.5) | 118 (55.4) |  |
| Overweight (23.0–25.0 kg/m^2^) | 284 (24.1) | 30 (21.1) | 119 (24.8) | 32 (20.0) | 45 (23.0) | 48 (22.5) |  |
| Obese(≥25.0 kg/m^2^) | 244 (20.7) | 29 (20.4) | 98 (20.5) | 35 (21.9) | 44 (22.4) | 41 (19.2) |  |
| **Education** |  |  |  |  |  |  | >0.999 |
| ≤Middle school | 143 (12.1) | 19 (13.4) | 59 (12.3) | 20 (12.5) | 25 (12.8) | 25 (11.7) |  |
| High school | 466 (39.6) | 53 (37.3) | 187 (39.0) | 62 (38.8) | 78 (39.8) | 86 (40.4) |  |
| ≥University | 569 (48.3) | 70 (49.3) | 233 (48.6) | 78 (48.8) | 93 (47.4) | 102 (47.9) |  |
| **Working status at survey** |  |  |  |  |  |  | 0.623 |
| Working | 648 (55.0) | 71 (50.0) | 265 (55.3) | 80 (50.0) | 113 (57.7) | 117 (54.9) |  |
| Not Working | 530 (45.0) | 71 (50.0) | 214 (44.7) | 80 (50.0) | 83 (42.3) | 96 (45.1) |  |
| **Marital status at survey** |  |  |  |  |  |  | 0.994 |
| Single | 94 (8.0) | 11 (7.7) | 42 (8.8) | 12 (7.5) | 12 (6.1) | 15 (7.0) |  |
| Married | 1039 (88.2) | 126 (88.7) | 416 (86.8) | 143 (89.4) | 179 (91.3) | 193 (90.6) |  |
| Divorced | 25 (2.1) | 3 (2.1) | 11 (2.3) | 3 (1.9) | 3 (1.5) | 3 (1.4) |  |
| Bereavement | 20 (1.7) | 2 (1.4) | 10 (2.1) | 2 (1.2) | 2 (1.0) | 2 (0.9) |  |
| **Monthly family income ($)** |  |  |  |  |  |  | 0.933 |
| ≤$2,000 | 167 (14.2) | 22 (15.5) | 69 (14.5) | 25 (15.6) | 25 (12.8) | 27 (12.7) |  |
| $2,000–$4,000 | 429 (36.4) | 59 (41.5) | 175 (36.7) | 64 (40.0) | 75 (38.3) | 78 (36.6) |  |
| >$4,000 | 582 (49.4) | 61 (43.0) | 233 (48.8) | 71 (44.4) | 96 (49.0) | 108 (50.7) |  |
| **Drinking status** |  |  |  |  |  |  | <0.001 |
| No | 412 (46.1) | 131 (92.3) | 93 (39.2) | 81 (50.9) | 104 (53.9) | 96 (55.8) |  |
| Yes | 482 (53.9) | 11 (7.7) | 144 (60.8) | 78 (49.1) | 89 (46.1) | 76 (44.2) |  |
| **Smoking status** |  |  |  |  |  |  | 0.046 |
| No | 655 (93.0) | 142 (100.0) | 259 (92.2) | 149 (93.1) | 114 (94.2) | 131 (93.6) |  |
| Yes | 49 (7.0) | 0 (0.0) | 22 (7.8) | 11 (6.9) | 7 (5.8) | 9 (6.4) |  |
| **Comorbidity (Yes)** | 322 (27.3) | 42 (29.6) | 131 (27.3) | 47 (29.4) | 56 (28.6) | 59 (27.7) | 0.985 |
| **Physical activity (Yes)** | 1175 (99.7) | 142 (100.0) | 479 (100.0) | 160 (100.0) | 195 (99.5) | 212 (99.5) | 0.659 |
| **Pathology stage** |  |  |  |  |  |  | 0.993 |
| 0 or CR (NRT) | 26 (2.9) | 1 (0.7) | 11 (3.0) | 6 (3.8) | 4 (2.7) | 4 (2.3) |  |
| I | 428 (47.1) | 74 (52.1) | 176 (47.6) | 80 (50.0) | 75 (50.7) | 81 (47.4) |  |
| II | 350 (38.5) | 51 (35.9) | 140 (37.8) | 56 (35.0) | 53 (35.8) | 67 (39.2) |  |
| III or Ⅳ | 104 (11.5) | 16 (11.3) | 43 (11.6) | 18 (11.2) | 16 (10.8) | 19 (11.1) |  |
| **Type of surgery** |  |  |  |  |  |  | 0.966 |
| Mastectomy with reconstruction | 33 (3.6) | 2 (1.4) | 12 (3.2) | 7 (4.4) | 7 (4.7) | 6 (3.5) |  |
| Mastectomy without reconstruction | 114 (12.6) | 19 (13.4) | 43 (11.6) | 20 (12.5) | 17 (11.5) | 23 (13.5) |  |
| Breast Conservation Surgery | 761 (83.8) | 121 (85.2) | 315 (85.1) | 133 (83.1) | 124 (83.8) | 142 (83.0) |  |
| **Chemotherapy (Yes)** | 649 (71.5) | 100 (70.4) | 267 (72.2) | 108 (67.5) | 100 (67.6) | 123 (71.9) | 0.981 |
| **Radiation therapy (Yes)** | 786 (86.6) | 125 (88.0) | 323 (87.3) | 136 (85.0) | 128 (86.5) | 148 (86.5) | 0.998 |
| **Hormone therapy (Yes)** | 661 (72.8) | 110 (77.5) | 265 (71.6) | 120 (75.0) | 118 (79.7) | 124 (72.5) | 0.783 |
| **Target therapy (Yes)** | 118 (18.4) | 16 (16.2) | 50 (18.8) | 20 (18.7) | 21 (21.4) | 20 (16.5) | 0.94 |

**Table S4.** Outcome data for training and validation set.

|  | **Training data set**  **(BIG-S cross-sectional study)** | **External validation set**  **(Best cohort)** |
| --- | --- | --- |
| **Survival period** |  |  |
| Overall | 3,026/6,265 (48.3%) | 573/1178 (48.6%) |
| Baseline (Pre-OP) | 1,335/1,980 (67.4%) | 100/142 (70.4%) |
| Under 1 year after surgery | 273/653 (41.8%) | 255/479 (53.2%) |
| Between 1 and 2 years after surgery | 497/1,265 (39.3%) | 79/160 (49.4%) |
| Between 2 and 3 years after surgery | 369/921 (40.1%) | 95/196 (48.5%) |
| Between 3 and 4 years after surgery | 246/682 (36.1%) | - |
| Between 4 and 5 years after surgery | 306/764 (40.1%) | 72/213 (33.8%) |

| **Table S5.** Machine learning results. | | | | | | |
| --- | --- | --- | --- | --- | --- | --- |
| **Survival period** | Metrics  Models | AUC | Accuracy | F1 score | Sensitivity | Specificity |
|  |  | Train (*SD*) | Train (*SD*) | Train (*SD*) | Train (*SD*) | Train (*SD*) |
| **Overall** | Deep Neural Network | 0.815 (0.023) | 0.753 (0.020) | 0.706 (0.027) | 0.750 (0.084) | 0.755 (0.069) |
|  | Gradient Boosting Machines | 0.816 (0.021) | 0.743 (0.024) | 0.702 (0.025) | 0.768 (0.080) | 0.727 (0.076) |
|  | XGBoost | 0.820 (0.021) | 0.762 (0.008) | 0.700 (0.026) | 0.708 (0.078) | 0.796 (0.051) |
|  | Light GBM | 0.819 (0.022) | 0.760 (0.011) | 0.700 (0.030) | 0.714 (0.082) | 0.790 (0.050) |
|  | CatBoost | **0.823 (0.020)** | **0.756 (0.018)** | **0.707 (0.019)** | **0.749 (0.067)** | **0.761 (0.060)** |
|  | Random Forest | 0.819 (0.022) | 0.759 (0.015) | 0.699 (0.037) | 0.721 (0.110) | 0.783 (0.077) |
| **Baseline**  **(Before surgery)** | Deep Neural Network | 0.822 (0.017) | 0.785 (0.017) | 0.834 (0.018) | 0.801 (0.043) | 0.752 (0.065) |
|  | Gradient Boosting Machines | 0.826 (0.019) | 0.755 (0.041) | 0.797 (0.043) | 0.724 (0.075) | 0.820 (0.057) |
|  | XGBoost | 0.822 (0.018) | 0.755 (0.028) | 0.800 (0.033) | 0.735 (0.066) | 0.794 (0.079) |
|  | Light GBM | 0.821 (0.017) | 0.748 (0.014) | 0.796 (0.018) | 0.730 (0.047) | 0.786 (0.069) |
|  | CatBoost | **0.835 (0.021)** | **0.774 (0.033)** | **0.817 (0.034)** | **0.753 (0.068)** | **0.815 (0.075)** |
|  | Random Forest | 0.826 (0.019) | 0.754 (0.028) | 0.799 (0.037) | 0.736 (0.087) | 0.791 (0.107) |
| **Under 1 years** | Deep Neural Network | 0.829 (0.050) | 0.792 (0.053) | 0.761 (0.045) | 0.791 (0.070) | 0.792 (0.106) |
|  | Gradient Boosting Machines | 0.859 (0.061) | 0.813 (0.046) | 0.776 (0.057) | 0.779 (0.088) | 0.837 (0.069) |
|  | XGBoost | 0.853 (0.057) | 0.813 (0.052) | 0.772 (0.067) | 0.761 (0.094) | 0.850 (0.070) |
|  | Light GBM | 0.854 (0.057) | 0.811 (0.049) | 0.770 (0.064) | 0.765 (0.104) | 0.845 (0.068) |
|  | CatBoost | **0.860 (0.056)** | **0.818 (0.055)** | **0.782 (0.068)** | **0.787 (0.098)** | **0.839 (0.079)** |
|  | Random Forest | 0.855 (0.049) | 0.808 (0.037) | 0.780 (0.044) | 0.817 (0.102) | 0.803 (0.091) |
| **Between**  **1 and 2 years** | Deep Neural Network | 0.757 (0.050) | 0.730 (0.049) | 0.676 (0.044) | 0.716 (0.079) | 0.738 (0.103) |
|  | Gradient Boosting Machines | **0.805 (0.055)** | **0.760 (0.037)** | **0.709 (0.056)** | **0.752 (0.121)** | **0.765 (0.086)** |
|  | XGBoost | 0.797 (0.055) | 0.746 (0.050) | 0.708 (0.062) | 0.790 (0.118) | 0.717 (0.100) |
|  | Light GBM | 0.797 (0.053) | 0.749 (0.050) | 0.704 (0.054) | 0.767 (0.118) | 0.737 (0.115) |
|  | CatBoost | 0.804 (0.051) | 0.765 (0.050) | 0.705 (0.072) | 0.722 (0.116) | 0.793 (0.082) |
|  | Random Forest | 0.798 (0.050) | 0.747 (0.047) | 0.701 (0.055) | 0.756 (0.081) | 0.741 (0.079) |
| **Between**  **2 and 3 years** | Deep Neural Network | 0.762 (0.041) | 0.724 (0.036) | 0.678 (0.026) | 0.729 (0.121) | 0.721 (0.131) |
|  | Gradient Boosting Machines | 0.796 (0.059) | 0.756 (0.053) | 0.695 (0.069) | 0.708 (0.147) | 0.788 (0.115) |
|  | XGBoost | 0.796 (0.054) | 0.751 (0.042) | 0.694 (0.054) | 0.707 (0.094) | 0.781 (0.074) |
|  | Light GBM | 0.796 (0.050) | 0.752 (0.050) | 0.698 (0.054) | 0.721 (0.139) | 0.773 (0.125) |
|  | CatBoost | **0.808 (0.051)** | **0.767 (0.037)** | **0.709 (0.054)** | **0.721 (0.117)** | **0.797 (0.086)** |
|  | Random Forest | 0.806 (0.055) | 0.771 (0.041) | 0.702 (0.065) | 0.689 (0.136) | 0.826 (0.101) |
| **Between**  **3 and 4 years** | Deep Neural Network | 0.759 (0.073) | 0.737 (0.069) | 0.659 (0.089) | 0.710 (0.136) | 0.752 (0.112) |
|  | Gradient Boosting Machines | 0.818 (0.035) | 0.787 (0.042) | 0.731 (0.047) | 0.800 (0.090) | 0.779 (0.083) |
|  | XGBoost | 0.794 (0.051) | 0.755 (0.061) | 0.702 (0.046) | 0.791 (0.100) | 0.733 (0.135) |
|  | Light GBM | 0.790 (0.052) | 0.759 (0.043) | 0.686 (0.052) | 0.736 (0.123) | 0.773 (0.106) |
|  | CatBoost | **0.820 (0.038)** | **0.783 (0.043)** | **0.723 (0.048)** | **0.792 (0.131)** | **0.777 (0.110)** |
|  | Random Forest | 0.810 (0.044) | 0.783 (0.039) | 0.708 (0.047) | 0.731 (0.091) | 0.812 (0.079) |
| **Between**  **4 and 5 years** | Deep Neural Network | 0.806 (0.040) | 0.760 (0.031) | 0.729 (0.034) | 0.807 (0.072) | 0.729 (0.066) |
|  | Gradient Boosting Machines | 0.821 (0.042) | 0.789 (0.031) | 0.744 (0.039) | 0.769 (0.099) | 0.804 (0.075) |
|  | XGBoost | 0.811 (0.041) | 0.789 (0.029) | 0.742 (0.028) | 0.758 (0.083) | 0.810 (0.080) |
|  | Light GBM | 0.809 (0.045) | 0.789 (0.034) | 0.741 (0.035) | 0.759 (0.117) | 0.810 (0.105) |
|  | CatBoost | 0.826 (0.045) | 0.793 (0.037) | 0.752 (0.033) | 0.782 (0.075) | 0.801 (0.088) |
|  | Random Forest | 0.822 (0.039) | 0.789 (0.033) | 0.749 (0.028) | 0.785 (0.073) | 0.792 (0.084) |

| **Table S6.** Shapley Additive Explanation value. | | | | | | |
| --- | --- | --- | --- | --- | --- | --- |
| Survival  period | Overall | | Baseline  (Before surgery) | | Under 1 years | |
| Rank | **Features** | **Shapley value** | **Features** | **Shapley value** | **Features** | **Shapley value** |
| 1 | Hopefulness | 0.2005 | Menopause symptoms | 0.2137 | Physical function | 0.3177 |
| 2 | Fatigue | 0.1853 | Emotional function | 0.1715 | Emotional function | 0.1995 |
| 3 | Side effects | 0.1449 | Side effects | 0.1473 | Side effects | 0.1989 |
| 4 | Physical function | 0.1210 | Social function | 0.1465 | Hopefulness | 0.1904 |
| 5 | Emotional function | 0.1178 | Fatigue | 0.1139 | Body image | 0.1004 |
| 6 | Role function | 0.1154 | Body image | 0.0733 | Role function | 0.0954 |
| 7 | Life purpose | 0.0928 | Drinking status | 0.0722 | Fatigue | 0.0923 |
| 8 | Social function | 0.0919 | Smoking status | 0.0715 | Dyspnea | 0.0886 |
| 9 | Menopause symptoms | 0.0816 | Physical function | 0.0659 | Menopause symptom | 0.0776 |
| 10 | Financial difficulties | 0.0781 | Life purpose | 0.0563 | Social function | 0.0758 |
| 11 | Monthly income | 0.0509 | Fear of recurrence | 0.0561 | Appetite loss | 0.0513 |
| 12 | Physical activity | 0.0483 | Hopefulness | 0.0360 |  |  |
| 13 | Pain | 0.0443 | Positive change | 0.0337 |  |  |
| 14 | Appetite loss | 0.0418 | Appetite loss | 0.0328 |  |  |
| 15 | Arm symptom | 0.0313 | Monthly income | 0.0326 |  |  |
| 16 | Dyspnea | 0.0282 | Sexual function | 0.0287 |  |  |

| **Table S6.** Shapley Additive Explanation value (continue). | | | | | | | | |
| --- | --- | --- | --- | --- | --- | --- | --- | --- |
| Survival  period | Between  1 and 2 years | | Between  2 and 3 years | | Between  3 and 4 years | | Between  4 and 5 years | |
| Rank | **Features** | **Shapley value** | **Features** | **Shapley value** | **Features** | **Shapley value** | **Features** | **Shapley value** |
| 1 | Fatigue | 0.2172 | Fatigue | 0.1819 | Fatigue | 0.1503 | Hopefulness | 0.2370 |
| 2 | Menopause symptoms | 0.1812 | Social function | 0.1532 | Emotional function | 0.1310 | Physical function | 0.1784 |
| 3 | Side effects | 0.1659 | Hopefulness | 0.1231 | Side effects | 0.1112 | Dyspnea | 0.1588 |
| 4 | Hopefulness | 0.1618 | Life purpose | 0.1013 | Physical function | 0.0925 | Financial difficulties | 0.1339 |
| 5 | Physical function | 0.1384 | Physical function | 0.0995 | Role function | 0.0911 | Monthly income | 0.1300 |
| 6 | Role function | 0.1348 | Role function | 0.0938 | Hopefulness | 0.0845 | Menopause symptoms | 0.1084 |
| 7 | Emotional function | 0.1225 | Financial difficulties | 0.0911 | Menopause symptom | 0.0800 | Side effects | 0.1028 |
| 8 | Nausea and vomiting | 0.0971 | Dyspnea | 0.0741 | Life purpose | 0.0749 | Emotional function | 0.1008 |
| 9 | Monthly income | 0.0966 | Side effects | 0.0685 | Financial difficulties | 0.0729 | Fatigue | 0.0901 |
| 10 | Fear of recurrence | 0.0657 | Sexual function | 0.0662 | Physical activity | 0.0728 |  |  |
| 11 | Life purpose | 0.0642 | Emotional function | 0.0630 | Breast symptom | 0.0640 |  |  |
| 12 | Pain | 0.0607 | Insomnia | 0.0527 | Social function | 0.0319 |  |  |
| 13 |  |  | Menopause symptom | 0.0480 | Pain | 0.0258 |  |  |
| 14 |  |  | Physical activity | 0.0463 |  |  |  |  |
| 15 |  |  |  |  |  |  |  |  |
| 16 |  |  |  |  |  |  |  |  |
|  | | | | | | | | |
